# Analysis of Newborn Hearing Screening Results in South Korea after National Health Insurance Coverage: A Nationwide Population-Based Study

**DOI:** 10.3390/ijerph192215052

**Published:** 2022-11-16

**Authors:** Kyu Young Choi, Su-Kyoung Park, Sun Choi, Jiwon Chang

**Affiliations:** Department of Otorhinolaryngology-Head and Neck Surgery, College of Medicine, Kangnam Sacred Heart Hospital, Hallym University, Seoul 07441, Republic of Korea

**Keywords:** hearing, newborn, neonatal screening, auditory brainstem response, otoacoustic emissions

## Abstract

Newborn hearing screening (NHS) has been covered by national health insurance since October 2018 in Korea. However, the results of the NHS are not reported due to the absence of a follow-up tracking system. This study analyzed the status and the predicted referral rates of NHS after the Korean national health insurance coverage by analyzing the National Health Insurance Service database in 2019 and 2020. The NHS coverage was 91.7% of total birth in 2019 and 92.1% in 2020. The predicted referral rate of NHS calculated by the duplicated NHS cases was 1.05% in 2019 and 0.99% in 2020. However, another predicted referral rate calculated by the number of diagnostic auditory brainstem responses (ABRs) performed was 1.44% in 2019 and 1.43% in 2020. The first NHS was performed within one day of birth for 96.5% of the babies and within three days of birth for 97%. However, diagnostic ABR was adequately performed within three months of birth for only 4.3%, while 82.3% performed the test after six months which delays appropriate intervention for hearing loss. National support such as national coordinators, follow-up tracking, and data management systems are needed for early hearing detection and intervention of newborns and infants in Korea.

## 1. Introduction

The incidence of permanent newborn hearing loss is between 1 and 3 for every 1000 births [1]. Moreover, almost half of the babies with congenital hearing loss are reported to be otherwise healthy without risk factors of hearing loss such as family history, neonatal intensive care unit stay, sepsis, meningitis, hyperbilirubinemia, use of ototoxic drugs, etc. [2,3,4,5]. Because delayed diagnosis and intervention may lead to permanent hearing loss that cannot be recovered later, early hearing detection and intervention (EHDI) of newborn hearing loss are most crucial for not only the development of language skills but also cognitive and social–emotional skills in these babies [3,6,7]. Since it is difficult to detect hearing difficulties in newborns, most developed countries offer universal newborn hearing screening (NHS) tests for all newborns [8]. In Korea, delayed marriages and low childbirth continued to increase. As a result, the Korean government decided to include newborn screening tests (hearing and congenital metabolic disorders) in the national health insurance in October 2018 as part of its fertility policy [9].

When the screening tests are not financially supported by national health insurance, some babies, especially in low-income families, may skip the test and be exposed to undetected hearing loss. However, the national health insurance fully supported the cost of NHS for all the children in Korea in October 2018, with the expectation of an increase in the performance of the NHS and, consequently, a decrease in the incidence of babies with hearing loss. It is recommended, in the international and Korean NHS guidelines, that screening tests be performed within one month of birth for all babies, confirmatory hearing tests within three months of birth for the referred cases, and intervention within six months of birth for the confirmed babies [2,3,10]. For the NHS in Korea, automated otoacoustic emissions (AOAE) or automated auditory brainstem response (AABR) tests are used at either the inpatient or the outpatient clinics. A diagnostic auditory brainstem response (ABR) is used for the referred cases in the screening tests to confirm hearing loss [10]. If the screening tests were performed during hospitalization, the government fully supports the screening fee. However, if the screening test is performed at the outpatient clinic, there is some co-payment depending on the income level, but this part is also supported by the Ministry of Health and Welfare [11]. The NHS screening pilot project for the 17 major cities in Korea from 2014 to 2018 reported significant differences in the performance of NHS and referral rates between the cities and the type of NHS, necessitating quality control and standardization by the whole country [9]. However, even after NHS tests were covered by national health insurance, tracking system and quality control are not yet implemented.

This study aimed to investigate the status and the predicted referral rates of NHS in Korea after the implementation of Korean national health insurance coverage of NHS by analyzing the National Health Insurance Service (NHIS) database.

## 2. Materials and Methods

### 2.1. Data Sources

This study was conducted by analyzing the nationwide big data from the Korean NHIS information database [12], which contains all data on health spending reimbursements and medical utilizations in Korea.

### 2.2. Study Design and Population

Since the NHS received support from the national health insurance in October 2018, this study analyzed newborns born between 1 January 2019 and 31 December 2020. Those who performed the 1st NHS within one year of their birth date and those reviewed by 31 August 2021, were enrolled in this study.

### 2.3. Main Outcomes and Measurement

The number of infants who underwent the NHS test under the national health insurance, NHS method (according to the hospitalization in 17 cities and provinces), the number of infants who performed the diagnostic hearing test (i.e., ABR), and the age (date) of the 1st screening and diagnostic ABR were evaluated based on the national test and surgical claim code classification.

We chose two methods for estimating the number of referred infants from the NHS test because the actual result of the NHS is not known from the NHIS information database. First, we estimated infants who did not pass NHS (i.e., “refer” result in NHS) by the duplicated cases in NHS (supposed to have performed 2nd NHS due to “refer” result in 1st NHS). As a second method, the number of ABR performed cases was used (supposed to have performed ABR due to the “refer” result in NHS).

### 2.4. Statistics and Ethics Approval

Statistical analyses were performed using SAS 9.3 software (SAS Institute Inc., Cary, NC, USA). The categorical data were presented by number (%). As a representative hospital, the study protocol was approved by the Institutional Review Board of Hallym University Kangnam Sacred Heart Hospital. The Institutional Review Board waived the need for informed consent because of the retrospective database nature of this study. All methods were performed according to the relevant guidelines and regulations.

## 3. Results

### 3.1. NHS Performance after National Insurance Coverage in Korea

The total number of live births in Korea was 306,273 in 2019 and 276,105 in 2020 (Table 1). The final NHS coverage rate compared to the total births in Korea was 91.7% and 92.1% in 2019 and 2020, respectively. Duplicated cases that had performed NHS at inpatient and outpatient clinics were included as one case for calculating the NHS coverage rate. In 2019, 97.2% had their tests in inpatient clinics with the expenses fully supported by the government, while 2.8% had outpatient clinic test with partial costs supported. However, the percentage of inpatient NHS increased to 97.7% in 2020. Compared to AOAE, AABR was used more for the NHS in Korea. However, the rate of AOAE usage was higher in the outpatient clinic compared to the inpatient clinic.

### 3.2. Regional NHS Status after National Insurance Coverage in Korea

The NHS performance was evaluated for the 17 major cities and provinces in Korea. Jeju island revealed the lowest NHS performance rate, while Seoul, the most populated city in Korea, showed the third lowest figure in 2019 (Table 2). However, the NHS rate in Jeju increased from 48.5% in 2019 to 70.6% in 2020.

### 3.3. Predicted Referral Rates Stratified by NHS Methods

Duplicated cases that had performed NHS both at inpatient and outpatient clinics were 2957 in 2019 and 2510 in 2020 (Table 1). The duplicated cases are considered those who had referred results in the inpatient clinic (mostly maternity clinics) that underwent retests in the outpatient clinic (those who passed the first NHS were considered normal and did not perform retest). The predicted referral rate calculated using the duplicated cases in NHS (i.e., the duplicate number divided by total NHS) was 1.05% in 2019 and 0.99% in 2020, respectively. While most cities in Korea revealed similar rates to the national mean referral rate, the predicted referral rate was highest in Gwangju and the lowest in Sejong in 2020 (Table 2).

### 3.4. Predicted Referral Rates Stratified by ABR Performance

Total number of infants who conducted diagnostic ABR tests each year was 4400 (inpatient 546 vs. outpatient 3854) in 2019 and 3948 (inpatient 519 vs. outpatient 3429) in 2020. The predicted referral rate calculated by the ABR’s performance, i.e., ABR’s number divided by the total NHS number, was 1.57% in 2019 and 1.55% in 2020, respectively.

### 3.5. Test Dates (Infant Ages) of the First NHS and ABR

Most NHS were performed within one day of birth (Table 3). More than 97% of infants performed their first NHS before seven days of birth, indicating that most NHS tests are carried out in inpatient settings. Among the 3662 infants who could confirm the time of the ABR test in 2020, only 4.3% of them performed the first ABR within three months of age, while most (82.3%) performed the test after six months of age (Table 4).

## 4. Discussion

The two representative screening tests for newborns in Korea are the NHS and the congenital metabolic disease screening tests [11]. The Korean government has supported congenital metabolic disease screening tests since 1997; however, NHS was started in 2007 as a pilot project that supported only low-income families (median income of 72% or less), both in the form of direct support from the Ministry of Health and Welfare not the national health insurance [9]. The others carried out NHS at their own expense until it was supported for all the babies from October 2018 by the national health insurance in Korea. The NHS pilot project supported only 17.6% of the babies born in Korea from 2014 to 2018 [9]. The rate increased to 91.7% in 2019 and 92.1% in 2020, showing increased performance of NHS after the national support in Korea. However, universal NHS in Korea was still not achieved, with 7.9% of newborns excluded from NHS in 2020.

The insurance coverage of NHS is beneficial not only to achieving EHDI but also to acknowledging the status of neonatal hearing for the national population. No national statistics on NHS in Korea could be reported before any project was performed by the government. While the national rate of actual neonatal hearing loss is not available, the estimated value can be achieved by analyzing the national database in this study. The mean national referral rate (1.5%) could be reported for the first time in Korea from the pilot project from 2014 to 2018 [2]. The results of the pilot project from 2014 to 2018 do not represent the whole babies in Korea because the project mainly included those in the low-income group. However, the results in this study are considered to be more accurate to the actual value because the financial support covered all cases. In this study, the predicted referral rate calculated by the duplicated NHS and the number of ABR were close to the previously reported value [9,13]. However, the values obtained by the number of ABR were higher due to the high-risk neonatal hearing loss patients who have been recommended to perform ABR despite the normal result in NHS [4].

Most 1st NHS was performed at the inpatient clinic when the babies were born, and most 2nd NHS and ABR were performed at the outpatient clinic after discharge from the hospital. Only 91.5% of NHS were performed at the inpatient clinic in the pilot project [2], however, the rate of inpatient clinic test significantly improved after the national insurance support. The reason for the variation in NHS coverage and refer rate among different regions in Korea might be the different existence of NHS devices in the local clinics, especially in rural areas. National support or an inter-hospital linkage system for such regions is needed. In particular, the NHS rate in Jeju was abnormally low in 2019 (48.5%), which increased to 70.6% in 2020. The education and promotion of NHS executed at the on-the-spot assessment of this rural area are considered to correct the abnormal value and increase the performance rate in Jeju, indicating the importance of the exposition of national support.

For diagnostic hearing loss tests, most ABRs (82.3%) in Korea were performed after six months of birth, showing delayed newborn hearing diagnostic tests in these patients. The 2.9% value within one day of birth group is considered as the misuse of ABR instead of the NHS in these cases. At the national level, it is necessary to provide guidance and education for the immediate performance of confirmatory testing for ‘refer babies’ in the NHS because early detection and intervention are crucial for conserving neonatal hearing loss [2,3,7,14]. For hearing loss management, the British government introduced the S4H system [15]. Centers for Disease Control and Prevention (CDC) and National Center for Hearing Assessment and Management (NCHAM) in the US, for a long time, have been managing programs called HiTRACK software and eSCREENER PLUS, using it as an educational and statistics site [16]. Each year, these countries analyze the statistics of neonatal hearing loss, publish them on sites, and hold workshops to share and educate local stakeholders and coordinators [17]. Considering the delay in diagnostic hearing loss tests, such effort is urgently needed by the government not only to support screening tests but also to have a management system at the national level.

The clinical significance of this study is that the government’s financial support of NHS offered not only the conservation of neonatal hearing but also enlightenment on the status and the statistics of the national NHS. The delayed time of ABR revealed in this study necessitates an effort to advance the timing of ABR tests for the NHS referred infants, such as parent education or financial support from the government. One limitation of this study is that the actual referral rate could be higher than the results presented here, considering those who missed ABR despite a refer result in NHS. Additionally, the COVID-19 situation that started in December 2019 may have affected the performance of the hearing tests.

## 5. Conclusions

Since Korea’s NHS test was applied to the national health insurance in 2018, the government’s NHS coverage rate has increased compared to the previous low-income class support project, but all newborns are yet to be screened. The status of NHS and predicted referral rate of NHS in Korea were discovered by analyzing the national data after the insurance coverage, for the first time to be reported in the literature. The analysis revealed that the referred babies in the NHS test were not adequately followed-up, and the diagnostic ABR test for confirming hearing loss was not implemented promptly. In Korea, where low fertility is a social problem, national support such as hearing loss coordinators, follow-up tracking, and data management systems are needed for the EHDI of all newborns and infants.

## Figures and Tables

**Table 1:** Yearly inpatient and outpatient newborn hearing screening status compared to total birth.

Year	Total Birth (t)	Inpatient NHS *n*, Infants (%)			Outpatient NHS *n*, Infants (%)			Final NHS (g) *n*, Infants (%) (g/t ×100)	Duplicated Cases (q)	Predicted Referral Rate (q/g ×100)
		AOAE (a) (a/c ×100)	AABR (b) (b/c ×100)	Total (c) (c/g ×100)	AOAE (d) (d/f ×100)	AABR (e) (e/f ×100)	Total (f) (f/g ×100)			
2019	306,273	12,935 (4.7)	260,037 (95.3)	272,972 (97.2)	1178 (14.9)	6718 (85.1)	7896 (2.8)	280,868 (91.7)	2957	1.05%
2020	276,105	11,379 (4.6)	236,959 (95.4)	248,338 (97.7)	703 (12.0)	5158 (88.0)	5861 (2.3)	254,199 (92.1)	2510	0.99%

NHS: newborn hearing screening; AOAE: automated otoacoustic emissions; AABR: automated auditory brainstem response.

**Table 2:** Newborn hearing screening status and the predicted referral rates in 17 Korean cities and provinces from 2019 to 2020.

Cities/Provinces	2019			2020		
	Total Birth	NHS % (*n*)	Predicted Referral Rate (*n*)	Total Birth	NHS % (*n*)	Predicted Referral Rate (*n*)
Seoul	54,851	85.6% (46,960)	0.96% (451)	48,786	84.9% (41,415)	1.16% (480)
Busan	17,226	93.2% (16,057)	0.85% (136)	15,302	93.6% (14,321)	1.03% (148)
Daegu	13,466	92.2% (12,417)	1.01% (125)	11,519	91.1% (10,489)	1.12% (117)
Incheon	18,501	90.0% (16,649)	1.38% (230)	16,181	88.1% (14,254)	1.34% (191)
Gwangju	8753	91.1% (7975)	3.71% (296)	7715	88.7% (6847)	2.92% (20)
Daejeon	8634	90.2% (7787)	0.64% (50)	7707	89.4% (6891)	1.02% (70)
Ulsan	7489	95.9% (7183)	2.07% (149)	6585	92.0% (6059)	1.82% (110)
Sejong	3643	89.7% (3266)	0.86% (28)	3334	93.5% (3117)	0.58% (18)
Gyeonggi	84,846	90.8% (77,050)	1.47% (1133)	78,996	89.4% (70,615)	1.42% (1003)
Gangwon	8134	83.9% (6828)	2.65% (181)	7675	82.8% (6358)	1.97% (125)
Chungbuk	9320	86.3% (8046)	1.45% (117)	8625	84.3% (7270)	0.89% (65)
Chungnam	13,390	93.4% (12,511)	2.15% (269)	12,142	91.1% (11,057)	1.60% (177)
Jeonbuk	9056	87.3% (7908)	2.40% (190)	8263	87.6% (7237)	1.58% (114)
Jeonnam	10,499	95.8% (10,056)	2.40% (241)	9413	92.0% (8661)	1.70% (147)
Gyeongbuk	14,382	92.8% (13,340)	1.13% (151)	12,762	91.5% (11,673)	1.65% (193)
Gyeongnam	19,232	94.5% (18,170)	1.50% (273)	16,863	92.8% (15,646)	1.62% (253)
Jeju	4451	48.5% (2157)	0.46% (10)	3955	70.6% (2791)	1.43% (40)

NHS: newborn hearing screening.

**Table 3:** Test date (infant age) of the 1st newborn hearing screening in 2020.

Test Date	Test No.		Test Rate/% (Total Birth)		Cumulative Test Rate %	
	AOAE	AABR	AOAE	AABR	AOAE	AABR
Within 1 day of birth	10,932	234,476	96.2	96.5	96.2	96.5
Within 2 days of birth	19	620	0.2	0.3	96.4	96.8
Within 3 days of birth	18	440	0.2	0.2	96.6	97.0
4–7 days of birth	55	1481	0.5	0.6	97.1	97.6
8–14 days of birth	77	1743	0.7	0.7	97.7	98.3
15–21 days of birth	103	1272	0.9	0.5	98.6	98.8
22–30 days of birth	78	803	0.7	0.3	99.3	99.2
31–60 days of birth	60	1108	0.6	0.5	99.9	99.6
After 60 days of birth	14	949	0.1	0.4	100	100
Total	11.359	242,892	100	100		

AOAE: automated otoacoustic emissions; AABR: automated auditory brainstem response.

**Table 4:** Test date (infant age) of the 1st diagnostic auditory brainstem response in 2020.

Test Date	Infant No.	Infant Percentage %
Within 1 day of birth	108	2.9
2–7 days	10	0.3
8–30 days	2	0.1
31–60 days	15	0.4
61–90 days	23	0.6
91–120 days	34	0.9
121–150 days	81	2.2
151–180 days	375	10.2
After 180 days	3014	82.3
Total	3662	100

## Data Availability

The excel data used to support the findings of this study were supplied by Su-Kyoung Park under license, and requests for access to these data should be made to S.K.P. ashock@daum.net.
